# Changing Arctic snow cover: A review of recent developments and assessment of future needs for observations, modelling, and impacts

**DOI:** 10.1007/s13280-016-0770-0

**Published:** 2016-03-17

**Authors:** Stef Bokhorst, Stine Højlund Pedersen, Ludovic Brucker, Oleg Anisimov, Jarle W. Bjerke, Ross D. Brown, Dorothee Ehrich, Richard L. H. Essery, Achim Heilig, Susanne Ingvander, Cecilia Johansson, Margareta Johansson, Ingibjörg Svala Jónsdóttir, Niila Inga, Kari Luojus, Giovanni Macelloni, Heather Mariash, Donald McLennan, Gunhild Ninis Rosqvist, Atsushi Sato, Hannele Savela, Martin Schneebeli, Aleksandr Sokolov, Sergey A. Sokratov, Silvia Terzago, Dagrun Vikhamar-Schuler, Scott Williamson, Yubao Qiu, Terry V. Callaghan

**Affiliations:** 1 FRAM – High North Research Centre on Climate and the Environment, Norwegian Institute for Nature Research (NINA), PO Box 6606, Langnes, 9296 Tromsø Norway; 2 Department of Ecological Sciences, Vrije Universiteit Amsterdam, De Boelelaan 1085, 1081 HV Amsterdam, The Netherlands; 3 Department of Bioscience, Arctic Research Centre, Aarhus University, Frederiksborgvej 399, 4000 Roskilde, Denmark; 4 NASA GSFC Cryospheric Sciences Laboratory, Code 615, Greenbelt, MD 20771 USA; 5 Goddard Earth Sciences Technology and Research Studies and Investigations, Universities Space Research Association, Columbia, MD 21044 USA; 6 State Hydrological Institute of Roshydromet, 23 Second Line V.O., St.Petersburg, Russia 199053; 7 International Centre for Science and Education “Best”, North-East Federal University, Yakutsk, Russia; 8 Climate Research Division, Environment Canada Ouranos, 550 Sherbrooke St. West, 19th Floor, Montreal, QC H3A 1B9 Canada; 9 Department of Arctic and Marine Biology, University of Tromsø, 9037 Tromsø, Norway; 10 School of GeoSciences, University of Edinburgh, Edinburgh, UK; 11 Institute of Environmental Physics, University of Heidelberg, Im Neuenheimer Feld 229, 69120 Heidelberg, Germany; 12 Department of Earth Sciences, Uppsala University, Villavägen 16, 75236 Uppsala, Sweden; 13 Department of Physical Geography and Ecosystem Science, Lund University, Sölvegatan 12, 223 62 Lund, Sweden; 14 Royal Swedish Academy of Sciences, PO Box 50005, 104 05 Stockholm, Sweden; 15 University Centre in Svalbard, PO Box 156, 9171 Longyearbyen, Norway; 16 Faculty of Life- and Environmental Sciences, University of Iceland, Sturlugata 7, 101 Reykjavík, Iceland; 17 Leavas Sámi Community, Box 53, 981 21 Kiruna, Sweden; 18 Arctic Research, Finnish Meteorological Institute, P.O. Box 503, 00101 Helsinki, Finland; 19 IFAC-CNR - Institute of Applied Physics “Nello Carrara”, National Research Council, Via Madonna del Piano 10, 50019 Sesto Fiorentino, FI Italy; 20 National Wildlife Research Centre, Environment Canada, 1125 Colonel By Drive, Ottawa, K1A 0H3 Canada; 21 Canadian High Arctic Research Station (CHARS), 360 Albert Street, Suite 1710, Ottawa, ON K1R 7X7 Canada; 22 Department of Physical Geography, Stockholm University, 106 91 Stockholm, Sweden; 23 Department of Earth Sciences, University of Bergen, 5020 Bergen, Norway; 24 Snow and Ice Research Center, National Research Institute for Earth Science and Disaster Prevention, 187-16 Suyoshi, Nagaoka, Niigata 940-0821 Japan; 25 Thule Insitute, University of Oulu, PO Box 7300, 90014 Oulu, Finland; 26 WSL Institute for Snow and Avalanche Research SLF, Flüelastrasse 11, 7260 Davos Dorf, Switzerland; 27 Arctic Research Station of Institute of Plant and Animal Ecology, Ural Branch, Russian Academy of Sciences, Labytnangi, Russia 629400; 28 Science Center for Arctic Studies, State Organization of Yamal-Nenets Autonomous District, Salekhard, Russia; 29 Arctic Environment Laboratory, Faculty of Geography, M.V. Lomonosov Moscow State University, Leninskie gory 1, Moscow, Russia 119991; 30 Institute of Atmospheric Sciences and Climate, National Research Council (ISAC-CNR), Corso Fiume 4, 10133 Turin, Italy; 31 Division for Model and Climate Analysis, R&D Department, The Norwegian Meteorological Institute, Postboks 43, Blindern, 0313 Oslo, Norway; 32 Department of Biological Sciences, University of Alberta, CW 405, Biological Sciences Bldg., Edmonton, AB T6G 2E9 Canada; 33 Institute of Remote Sensing and Digital Earth, Chinese Academic of Science, Beijing, 100094 China; 34 Group on Earth Observations, Cold Regions Initiative, Geneva, Switzerland; 35 Department of Animal and Plant Sciences, University of Sheffield, Sheffield, S10 2TN UK; 36 National Research Tomsk Stated University, 36, Lenin Ave., Tomsk, Russia 634050

**Keywords:** Climate change, Ecosystem services, Human health, Societal costs, Indigenous, Snow

## Abstract

Snow is a critically important and rapidly changing feature of the Arctic. However, snow-cover and snowpack conditions change through time pose challenges for measuring and prediction of snow. Plausible scenarios of how Arctic snow cover will respond to changing Arctic climate are important for impact assessments and adaptation strategies. Although much progress has been made in understanding and predicting snow-cover changes and their multiple consequences, many uncertainties remain. In this paper, we review advances in snow monitoring and modelling, and the impact of snow changes on ecosystems and society in Arctic regions. Interdisciplinary activities are required to resolve the current limitations on measuring and modelling snow characteristics through the cold season and at different spatial scales to assure human well-being, economic stability, and improve the ability to predict manage and adapt to natural hazards in the Arctic region.

**Electronic supplementary material:**

The online version of this article (doi:10.1007/s13280-016-0770-0) contains supplementary material, which is available to authorized users.

## Introduction

Snow is a critically important element of the Arctic and is rapidly changing due to climate warming (Callaghan et al. 2011). Snow cover, stratigraphy, and physical characteristics are naturally changing throughout the seasons but are likely to be affected by climate warming with unexpected impacts for ecosystems and society. For example, Arctic snow-cover duration is decreasing rapidly (~3–5 days/decade), particularly due to earlier spring melt (20 %/decade) and later onset of snow cover (Derksen et al. 2015). However, the Eurasian Arctic region has experienced larger declines in the duration of the snow-covered period (12.6 days), i.e. prolonged vegetation growing season, compared to the North American Arctic region (6.2 days) between 1982 and 2011 (Barichivich et al. 2013). In addition, climate warming increases the potential for unseasonal thaws, early snowmelt, and rain-on-snow events (ROS) (Liston and Hiemstra 2011). These changes impact snow properties and runoff (Semmens et al. 2013), which in turn affect Arctic ecosystems and societies (Meltofte 2013; Cooper 2014; Hansen et al. 2014). However, changes in snow properties are not uniform across the Arctic and affected processes operate/respond at different temporal and spatial scales. Moreover, the various disciplines working on snow measure and evaluate its properties at different temporal and spatial scales. Therefore, there are potential mismatches on the availability and requirements of snow data between snow scientists, modellers, ecologists, and sociologists.

To address these issues, an interdisciplinary workshop was held to develop a road map to improve measurement, modelling, and prediction of changing snow characteristics and to collate developments in the field since the “Snow Water Ice and Permafrost in the Arctic” assessment of 2011(Callaghan et al. 2011). This paper builds on the results presented at the workshop and presents an overview of recent developments in studies of changing Arctic snow cover and its consequences.

## Understanding the impacts of changing snow conditions on societies and ecosystems

### Economy, human health, and well-being

**Fig. 1:**
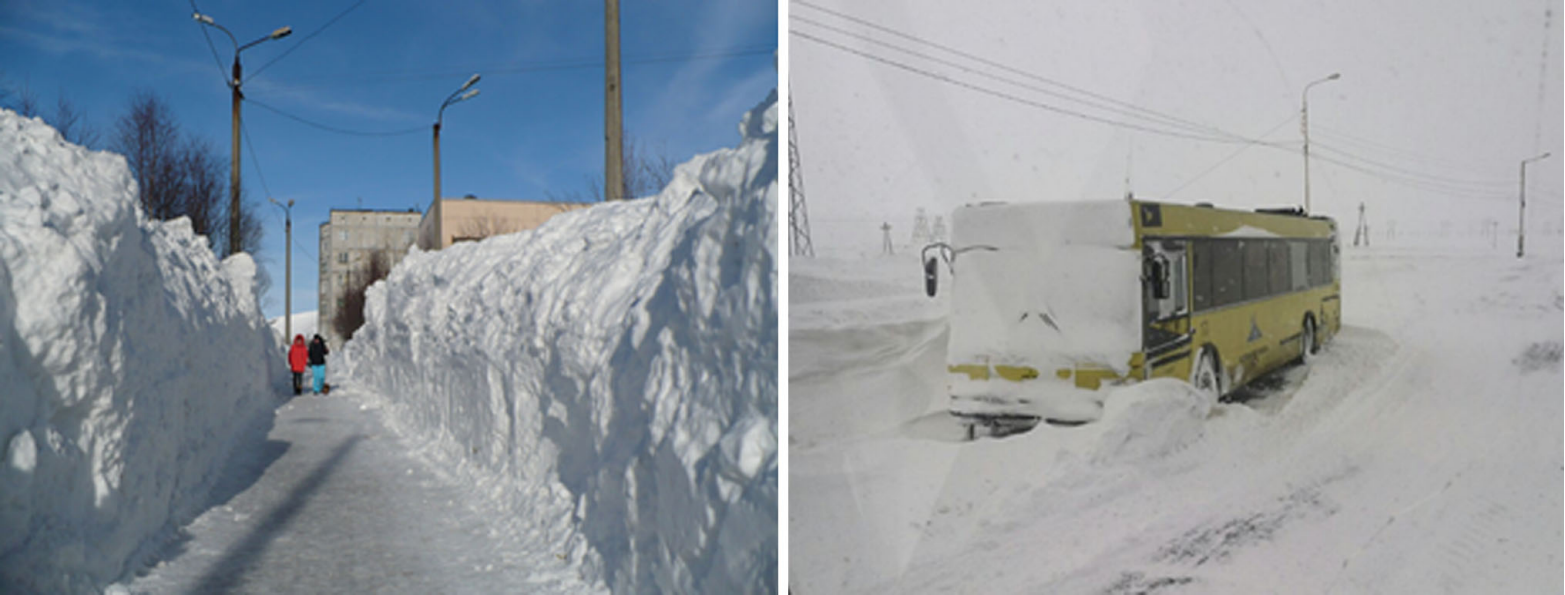
Increases in heavy snowfall affect the function of cities above the Arctic Circle. Snow clearance (*left*) has economic costs, whereas lack of snow clearance (*right*) can perhaps have even greater costs (*left* Kirovsk and *right* Norilsk: photos M.N. Ivanov)

The direct impact of snow temporal and spatial variability on economic development of the Arctic has to our knowledge not been comprehensively evaluated and quantified. Such a study would need to take into account among others: Snow clearing costs of transportation routes (Hanbali 1994; Riehm and Nordin 2012) (Fig. 1), which varies annually and is complicated by extreme snowfalls (Borzenkova and Shmakin 2012). The prevention of freezing damage to water pipes and drainage systems (Bjerke et al. 2015). Associated risks to winter-crops and forestry production due to changes in snow-season duration (Hanewinkel et al. 2011; Krenke et al. 2012), increased frequency of desiccation, exposure to snow moulds (Matsumoto and Hoshino 2009), and encasement in ground ice (Bjerke et al. 2014, 2015). Furthermore, ice-based construction procedures relying on firn-ice (e.g. winter roads) can be affected (Sosnovsky et al. 2014). Seasonal snow conditions are crucial for the way of life of indigenous people and local residents for reindeer herding practices and access to hunting grounds (Riseth et al. 2011), harvest yields of cultivated and wild berries (Bokhorst et al. 2011; Niemi and Ahlstedt 2012), and game animals (Stien et al. 2012; Hansen et al. 2013). Snow-season duration and snow-cover depth also affect the economy through changes in the magnitude and timing of spring runoff and floods. In Siberia, the frequency of dangerous river ice jams and spring river flooding events are increasing (Popova 2011; Semenov 2013), while decreased snow precipitation will affect the water supply for aquatic ecosystems, forestry, and agriculture (Jeelani et al. 2012; Clarke et al. 2015).

The increasingly wetter and milder Arctic climate can lead to increased frequency of avalanches threatening growing populations and infrastructure (Eckerstorfer and Christiansen 2012; Qiu 2014). When comparing snow avalanche risk assessments between regions, losses are often associated with an increase in land use, population density, and economic activities (Shnyparkov et al. 2012). Healthcare costs can rise due to increasing occurrence of bone fractures resulting from unusual snow and ice conditions (Bjerke et al. 2015). Snow can also become a health issue when supporting biological pathogens (Biedunkiewicz and Ejdys 2011; Shen and Yao 2013; Simon et al. 2013; Ejdys et al. 2014). The impacts of changing snowmelt dynamics on snow-pathogens for humans, livestock, and agriculture are unclear (Parham et al. 2015).

### Ecosystems

Snow cover is an important determinant of community and ecosystem structure in polar regions (AMAP 2011) and winter temperatures are increasing in the Arctic more than those during summer (Walsh 2014). However, impacts of changing winter climate and snow regimes have received much less attention compared to the effects of climate change during summer. Different aspects of the snowpack play crucial roles in ecosystem processes and the life of Arctic organisms (e.g. Cooper 2014). Relevant snowpack characteristics include thermal insulation, snow depth, microstructure, temporal changes of these aspects, as well as snow-cover duration, all of which have been shown to be affected by climate change, with important consequences for Arctic ecosystems (AMAP 2011).

#### Terrestrial ecosystems

**Fig. 2:**
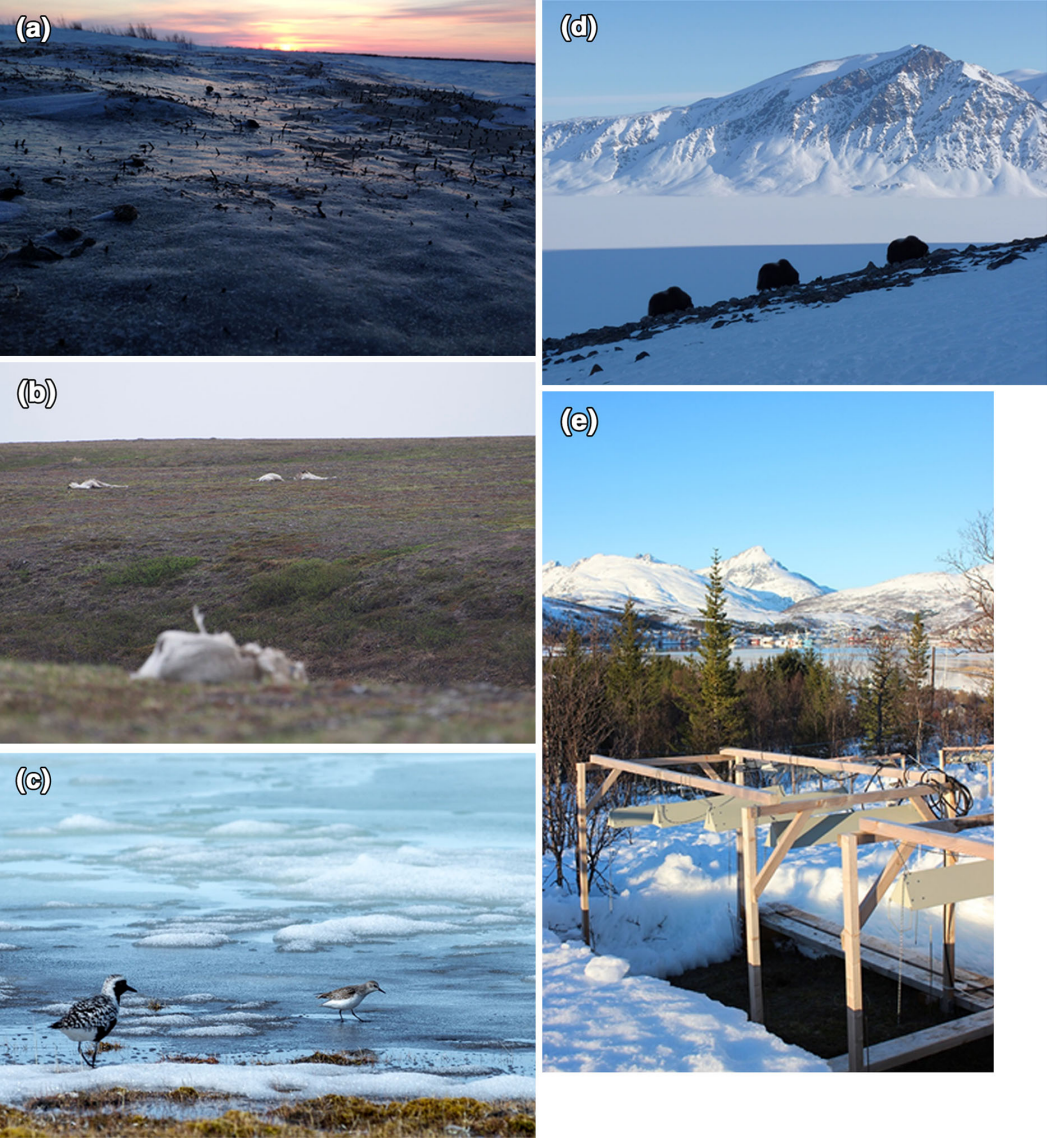
Examples of changing snow conditions in terrestrial ecosystems: **a** Vegetation captured in ice layer following rain-on-snow event leading to **b** mortality among reindeer (Yamal Russia) and **c** delayed breeding of Black-bellied Plover (*Pluvialis squatarola*) (Southampton Island, Nunavut, Canada); **d** Muskoxen (*Ovibos moschatus*) grazing at high elevation to find snow-free patches during spring 2012, Zackenberg in Northeast Greenland; **e** Experimental simulation of extreme winter warming near Tromsø (Norway). Photos **a** and **b** Aleksandr Sokolov, **c** K. Young, **d** S. Højlund Pedersen, and **e** S. Bokhorst

Snow acts as an insulating blanket against freezing Arctic temperatures for many organisms. Snow is also a major determinant of the mosaic of ecological communities through its uneven landscape distribution and the influence of snowmelt-driven spring flooding on wetland communities. Changes in snow quantity, quality, and seasonality can, therefore, result in changes in the distribution and composition of Arctic communities with resulting effects on their many inherent ecological processes, functions, and feedbacks. Extreme weather events (unseasonal warm temperatures and ROS see Fig. 2) can cause complete loss of snow cover, changes in the snow stratigraphy, snow hardness, and formation of ice layers with great impacts on plants (Bokhorst et al. 2011; Preece et al. 2012), herbivores (Bartsch et al. 2010; Ims et al. 2011; Stien et al. 2012; Bilodeau et al. 2013), soil organisms and CO_2_ fluxes (Bokhorst et al. 2012, 2013), and agriculture (Bjerke et al. 2014, 2015). However, species responses to extreme weather events and snowmelt are dependent on the timing of events (Bokhorst et al. 2010, 2011), while the mechanisms behind species responses are unclear (Rumpf et al. 2014; Bowden et al. 2015) and processes are often inferred based on indirect correlative information (e.g. Ims et al. 2011). Furthermore, changing snow conditions can have wide-ranging indirect effects mediated by ecological interactions. For instance, shrub growth affects snow accumulation which in turn influences soil temperatures and ecosystem process rates (Myers-Smith and Hik 2013) highlighting the importance of interactions between vegetation structure and snow properties. Snow-induced changes in mortality and dynamics of reindeer and lemming (Hansen et al. 2013) affect predator populations (Schmidt et al. 2012) which in turn may shift to alternative prey (McKinnon et al. 2013; Nolet et al. 2013). These examples highlight the need to identify critical periods when species and ecosystems are vulnerable to winter climate change, especially with regard to periods of snowpack build-up, ROS and ground icing, and spring snowmelt.

Aside from the species-specific and ecosystem responses to changing snow conditions, there is a major research challenge in linking the predictions of snow changes to the scales that are relevant for the organisms or ecosystem that is being studied (Table 1). Specifically, there is a need for accurate predictions of the build-up and change in the snow stratigraphy across scales of a few square metres to landscapes covering several km^2^.

**Table 1:** Overview of the various expected changes in snow conditions, affected groups of organisms, processes, or activities and the modelling requirements that are required to predict their occurrence in the near future. The different affected groups, processes, and/or activities have different spatial and temporal extent and resolution; hence models are required to resolve these specific spatial and temporal dimensions

Changes in climate and snow	Affected groups/processes	Modelling requirements to predict these changes	Scale
Temperature variability under the snow (snow insulation)	Soil organisms, dwarf shrubs, cryptogams	Snow depth, snow density, snow type, stratigraphy, and temporal evolution of these through the cold season	0–1 m^2^
	Ecosystem CO_2_ fluxes		0–1 m^2^
	Shrubs and trees		1–10 m^2^
Ice-layer formation	Humans, sub-Arctic agroecosystems, vegetation, small rodents, reindeer, and species depending on them through direct or indirect trophic interactions	Timing, duration/longevity, compactness, and spread of (ground) ice formation across the landscape, in urban areas, and on transportation infrastructure (roads, airports, culverts)	1–10 m^2^ and >km^2^
Avalanche risk	Society, infrastructure, large grazers, and mountainside vegetation, especially trees	Snow stratigraphy/stability through the cold season	100 m^2^
Snow accumulation	Infrastructure/society, water supply, large grazers and flooding risk	Snow depth, snow water equivalent, timing of heavy snowfall events, and snow (re-)distribution by wind	<100 m^2^
Snow-cover duration and timing	Agriculture, freshwater ecosystems, terrestrial ecosystems, energy use, northern food security, transportation, and recreation	Snow depth, timing of snow deposition and snowmelt, and resultant sea ice melt out	<100 m^2^

#### Freshwater systems

Snow on lake and river ice affects the temperature and light transmission to the underlying ice and water. Changes in the snowpack can therefore affect the freezing regime, having consequences for the freshwater ecosystem with feedbacks to habitat structure, food availability, and survival of species (Prowse and Brown 2010; Prowse et al. 2011; Surdu et al. 2014). For shallow waters (<3 m) and wetlands, the timing and duration of ice defines the open water, productive period and limits the active state of aquatic organisms by freezing to the bottom. Winter-dormancy allows species to survive such frozen conditions but the breaking of winter-dormancy depends on the photoperiod and temperature (Dupuis and Hann 2009) which is affected by the snow cover. Particularly the formation of ‘white ice’, formed when the snowpack exceeds the buoyance of the ice, affects the light transfer to the water column below (Dibike et al. 2012). Changing snow conditions affecting freshwater freezing and melting conditions may cause mismatches for organisms in terms of when winter-dormancy ends compared to peak food availability. Ecosystem phenology associated with ice and snow cover in freshwater systems is an area that needs more research.

Spring snowmelt is also an important conduit for transporting organic matter from the land into rivers and lakes. This pulse of organic matter into freshwater affects the clarity (light attenuation), nutrient and carbon cycling, primary productivity, and overall food web dynamics of aquatic ecosystems (Ask et al. 2009; Rautio et al. 2011). Furthermore, dissolved and suspended concentrations of metals are highest in rivers and lakes during the spring freshet (Holemann et al. 2005) indicating that the snowpack acts as a reservoir for contaminants that are released as a pulse (Douglas et al. 2012). The timing of mercury (Hg) runoff, for example, is greatly affected by the spatial variability in hill-slope flow paths and the magnitude of snowmelt inputs (Haynes and Mitchell 2012) indicating that predictions of mercury runoff in water streams need to be developed at small scales and that up-scaling will be challenging.

#### Sea ice and snow

Variations in snow-covered sea ice affect the Earth’s climate by affecting ocean–atmosphere interactions. Snow cover on top of sea ice has a high albedo that dominates the surface solar energy exchange, and a changing thermal conductivity that regulates ice/atmosphere heat transfer that greatly modifies the sea ice thermodynamic processes. The snow cover also modifies surface roughness with implications for the ice/air drag coefficient and sensible and latent heat fluxes. Snow depth and snow properties (e.g. thermal conductivity and density) on sea ice are thus of crucial importance, and must be accurately retrieved on a large scale.

Snow across sea ice influences algal communities with thin snow cover promoting productivity in the ocean (Alou-Font et al. 2013). This suggests that reduced snow precipitation or quicker melt out may promote higher primary production underneath sea ice with potential positive impacts higher up the food chain. Conversely, snow-cover removal from the sea ice surface can inhibit spring growth of Arctic ice algae through physiological and behavioural effects (Lund-Hansen et al. 2014).

### Teleconnections and snow cover in Arctic amplification

Research has been dedicated to investigate the linkages between the changing Arctic snow cover and tropospheric processes (Cohen et al. 2014) and the impacts of Arctic amplification to temperature variability at low and high latitudes (Francis and Vavrus 2012; Screen 2014). Declining terrestrial spring snow cover in the Arctic is contributing to Arctic amplification (Serreze and Barry 2011; Matsumura et al. 2014). Changing snow on freshwater systems affect local climate conditions (Rouse et al. 2008; Brown and Duguay 2010). Observations of Arctic sea ice reduction in autumn are shown to be causing cold extremes (e.g. additional snowfall) in mid-altitude and northern continents/sub-Arctic areas (Cohen et al. 2013; Tang et al. 2013). Arctic amplification depends on heat-transport from lower latitudes but local factors on surface warming is still a matter of debate because it is difficult to isolate local forcings from simultaneously occurring external forcings and feedbacks (Screen and Simmonds 2012). Furthermore, high-latitude responses in the multiple types of forcing between models were broad, making it difficult to define the particular causes of Arctic temperature amplification (Crook et al. 2011). Improved process understanding, additional Arctic observations, and further modelling efforts in collaboration with observation data are required to elucidate the teleconnections with the Arctic (Cohen et al. 2014).

## Observations of changing snow conditions

Quantifying snow-cover extent, thickness, and specific snow characteristics in the Arctic is challenging mainly due to the inclement weather conditions, polar night, and redistribution of snow by wind. In addition, the limited Arctic snow-observation stations challenge the up-scaling process to larger regions. However, there is a great need for accurate snow data at different spatial and temporal resolutions to address the challenges of changing snow conditions. We present an overview of recent advances in methods for quantifying and monitoring snow variables, and a summary of widely used ground-based snow observational methods is presented in Table 2. In addition, we indicate data/knowledge gaps where progress is required in terms of spatial and temporal resolution of snow variables.

**Table 2:** Overview of observation methods in quantifying various snow parameters

Target parameter(s)	Method(s)	Reference(s)
	Destructive ground-based snow observations	
Snow depth	Simple (avalanche) or semi-automated probes (e.g. MagnaProbe)	e.g. Sturm et al. (2006)
Specific surface area (SSA) (i.e. the surface area of ice per unit mass)	Near-infrared photography and infrared reflectance methods	e.g. Matzl and Schneebeli (2006), Gallet et al. (2009) Arnaud et al. (2011), and Montpetit et al. (2012)
Penetration resistance and deviation of snow density, grain parameters, and SSA.	SnowMicroPen (Highly resolved measurements (250 measurements/mm)	Schneebeli and Johnson (1998) and Proksch et al. (2015)
Snowfall/new snow	Snow board (i.e. new-snow observations are being conducted by placing a board (snow board) on the snow surface and revisiting it every 24 h to read the additional snow height	e.g. Fierz et al. (2009)
Liquid water content in snow	‘Denoth capacity probe’ or ‘Finnish Snow Fork’ (e.g. used to deriving dielectric/conduction properties of the snow)	Denoth (1994) and Sihvola and Tiuri (1986)
	Non-destructive ground-based snow observations	
Snow depth	Acoustic snow-depth sensors, ultrasonic methods, lasers, manual readings at stakes, and automatic readings utilizing time-lapse cameras	
Snow density and snow bulk liquid water content	Upward-looking ground penetrating radar (upGPR) Combination of upGPR with buried GPS sensors (allows for direct conversion for density, SWE and liquid water content) Time domain reflectometer (TDR)	e.g. Mitterer et al. (2011), Avanzi et al. (2014), Heilig et al. (2015), Schmid et al. (2014, 2015), and Stacheder (2005)
Snow water equivalent (SWE)	Snow pillows or snow scales weigh the mass of the snowpack above the sensors and convert this to SWE	
Snow albedo	Net radiometer	e.g. Michel et al. (2008)
Snow-cover fraction	Derived from hourly-daily digital photos acquired from automatic time-lapse digital cameras installed in terrestrial areas, e.g. near glaciers and ice fields	e.g. Bernard et al. (2013)
Avalanche hazard and activity	Seismic sensor	Reiweger et al. (2015)
	Infrasound arrays	e.g. Van Herwijnen and Schweizer (2011), Havens et al. (2014)

### Overview of recent advances in methods and findings in Arctic snow monitoring

#### Ground-based snow-depth monitoring

Several well-known methods for measuring snow depth exist (Table 2). Recent developments in snow-depth measurements include remote sensing methods that enable an objective monitoring of spatial distributions of snow depth. These methods include polarimetric phase differences (Leinss et al. 2014), ground-based laser scans (Deems et al. 2013), and electromagnetic wave technology (e.g. Koch et al. 2014; McCreight et al. 2014).

#### Spaceborne snow-cover monitoring

Snow-cover has high spatial and temporal variability and satellites provide observations at the hemispherical scale. Both passive and active remote sensing methods are used with sensors operating in the visible and microwave domains. Visible sensors observe snow-surface properties (with solar illumination, in cloud-free conditions), and are used for mapping snow-cover extent (e.g. Hall et al. 2002, 2006). Microwave sensors are sensitive to snow properties, and operate independently from solar illumination with a weak sensitivity to the atmosphere. The main limitation of using microwave radiometers is the coarse resolution (i.e. tens of kilometres), whereas radars lack the appropriate frequencies. Existing radar sensors, which can provide information on snow-cover with fine resolution, are able to work only in the presence of wet snow.

#### Snow water equivalent (SWE)

Satellite algorithms have been developed to monitor SWE at the hemispherical scale since the 1980s (e.g. Kelly 2009). In the early 2000s, surface-based Frequency-Modulated Continuous-Wave (FMCW) radar measurements were used to estimate SWE to within 5 % (Marshall et al. 2005). Furthermore, fixed radars installed underneath or above the snow cover have been used for deriving snow depth, density, bulk liquid water content, and for deriving SWE (Heilig et al. 2009; Schmid et al. 2014) and allow monitoring of the temporal evolution of the overlying snow. In addition, recent advances in SWE quantification have shown the benefit of combining passive microwave radiometer and ground-based synoptic weather station observations to provide robust information on hemispherical scale (Takala et al. 2011). Mobile measurements allow for monitoring spatial differences in SWE or liquid water content but only provide snapshots in time. Hence, there are major challenges to compare satellite-derived information with ground-based in situ data. In addition, further development on sensors for satellites and aircrafts is necessary including new technologies for data interpretation together with up-scaling methods for temporal continuous point measurements. Further investigations are required to convert satellite observations into accurate SWE retrievals and remote sensing of SWE is currently restricted to flat areas thereby excluding mountains.

#### Snow microstructure (grain size, snow-specific surface area) and liquid water content (LWC)

**Fig. 3:**
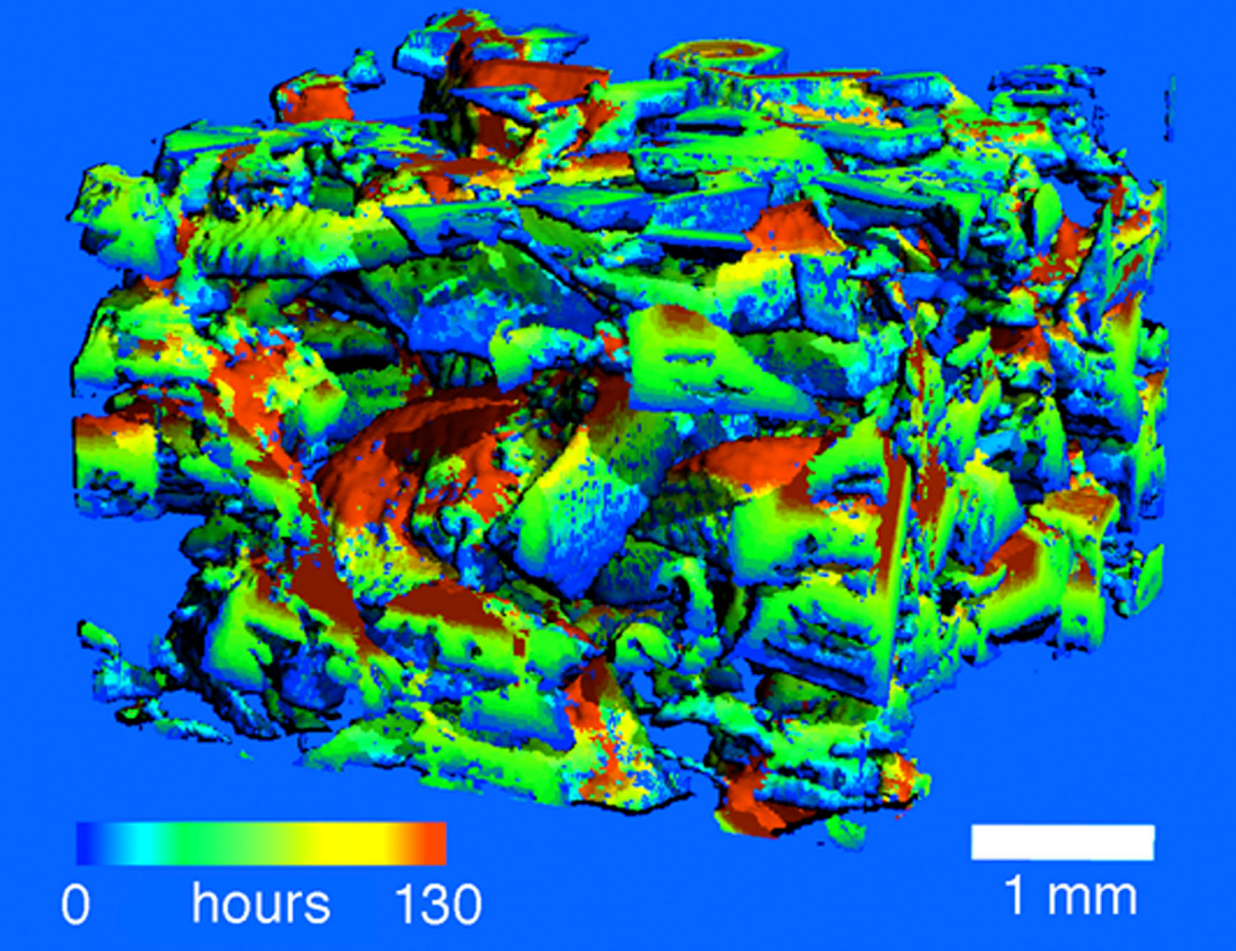
Age distribution of ice in a depth hoar sample from a laboratory experiment. The depth hoar sample has been exposed to typical temperature gradients of an Arctic snowpack (5°K snow temperature increase per 10 cm depth). Depth hoar recrystallizes completely and the oldest parts of the sample are just 5-days old ice (*dark red*), although the snow was made 28 days before (M. Schneebeli, WSL-SLF, unpublished)

Snow microstructure is complex, but can be characterized by snow-specific surface area (SSA). SSA controls the snow albedo and is a more objective measure of snow’s complexity than grain size. SSA typically decreases with time with a rate depending on temperature and the shape of the initial snow grain (Hachikubo et al. 2014). SSA measurements have been successfully conducted in the field using near IR methods (Gallet et al. 2009; Arnaud et al. 2011; Montpetit et al. 2012). The SnowMicroPen, which uses highly resolved penetration resistance (250 measurements/mm), can be used to quantify snow density, grain size, and SSA (Proksch et al. 2015). Time-lapse X-ray micro-tomography methods provide a 3D reconstruction of the snow structure (Pinzer et al. 2012) and enable visualization of the recrystallization distribution on depth hoar crystals through time (Fig. 3). Recent development of SSA measurements led to implementation of SSA parametrizations in snow evolution modelling (Carmagnola et al. 2014). Advances in thermal and short IR remote sensing allow for determining surface snow types and surface temperature (Hori et al. 2014).

In snow hydrology, the onset and the total amount of runoff are essential for flood and reservoir management, and impact on terrestrial ecosystems. The change in dielectric permittivity of snow during melt highly influences remote sensing data from microwave to infrared, allowing us to monitor the extent of surficial melt (e.g. Steffen et al. 2004). Modelling of LWC and snowpack runoff is still very challenging and water transport schemes like a multi-layer bucket model or Richards equation underestimate observed maximum LWC in the course of a season (Heilig et al. 2015). LWC retention in the snow is important to improve modelled runoff performance (Essery et al. 2013; Heilig et al. 2015).

#### Snow-surface albedo and light-absorbing impurities

Impurities in the snowpack can affect the snowmelt rates through decreased surface albedo. Such light-absorbing snow impurities include organic carbon, mineral dust, and micro-organisms (Langford et al. 2010), and can be quantified in manually collected snow samples and by reflectance measurements. Algal communities have been associated with glacial melt and reducing snow-surface albedo (e.g. Tedesco et al. 2013; Lutz et al. 2014). Similar responses to deposits of black carbon (BC) on the snow surface are shown to cause accelerating snowmelt rates in Alaska, Norway, and Greenland (Doherty et al. 2013). Particle size of snow impurities can be used to identify their source and have been linked to peripheral snow-free areas or locations with early snowmelt and fires (Aoki et al. 2014; Dumont et al. 2014). A decreasing snow-cover extent may play a major role in the surface mass balance of Arctic ice bodies.

#### Snow on sea, lake, and river ice

Snow cover on sea ice influences the Earth’s climate and biology in the ocean. The only current snow-depth-on-sea-ice algorithm that uses satellite data is based on passive microwave observations (Cavalieri et al. 2012; Brucker and Markus 2013). Since 2009, NASA has supported the airborne Operation IceBridge mission, which operates multiple radars to retrieve snow depth on sea ice (Kurtz et al. 2013; Panzer et al. 2013). Recent work on IceBridge data and from drifting ice station indicates a substantial thinning of the snowpack in the western Arctic and in the Beaufort and Chukchi seas (Webster et al. 2014). This thinning is negatively correlated with the delayed onset of sea-ice freeze-up during autumn. Thin snowpack and sea ice increase the heat flux between the ocean and atmosphere with potential feedbacks for the Earths’ climate but are not thoroughly investigated. Although snow on lake ice has major implications for lake ecology, ice thickness, and the local climate (Brown and Duguay 2010), studies on these systems appear to be under-represented in the literature (Cheng et al. 2014; Duguay et al. 2015). Furthermore, there is currently little focus on quantifying changes in lake-ice snow cover. The most recent progress in remote sensing is summarized in Duguay et al. (2015).

#### Avalanche detection

Recent advances in avalanche detection include the use of seismic sensors and infrasound arrays (Table 2). Furthermore, Synthetic Aperture Radar (SAR), e.g. Radarsat-2, TerraSAR-X, and Cosmo-Skymed, have been shown useful in detecting avalanche activity. Especially, the SAR data properties as the spatial resolution (2–3 m), high temporal resolution (2–5 days), and their application during cloudy conditions make them ideal for this purpose (Caduff et al. 2015).

### Indigenous knowledge: Sámi snow observational methods and terminology

**Fig. 4:**
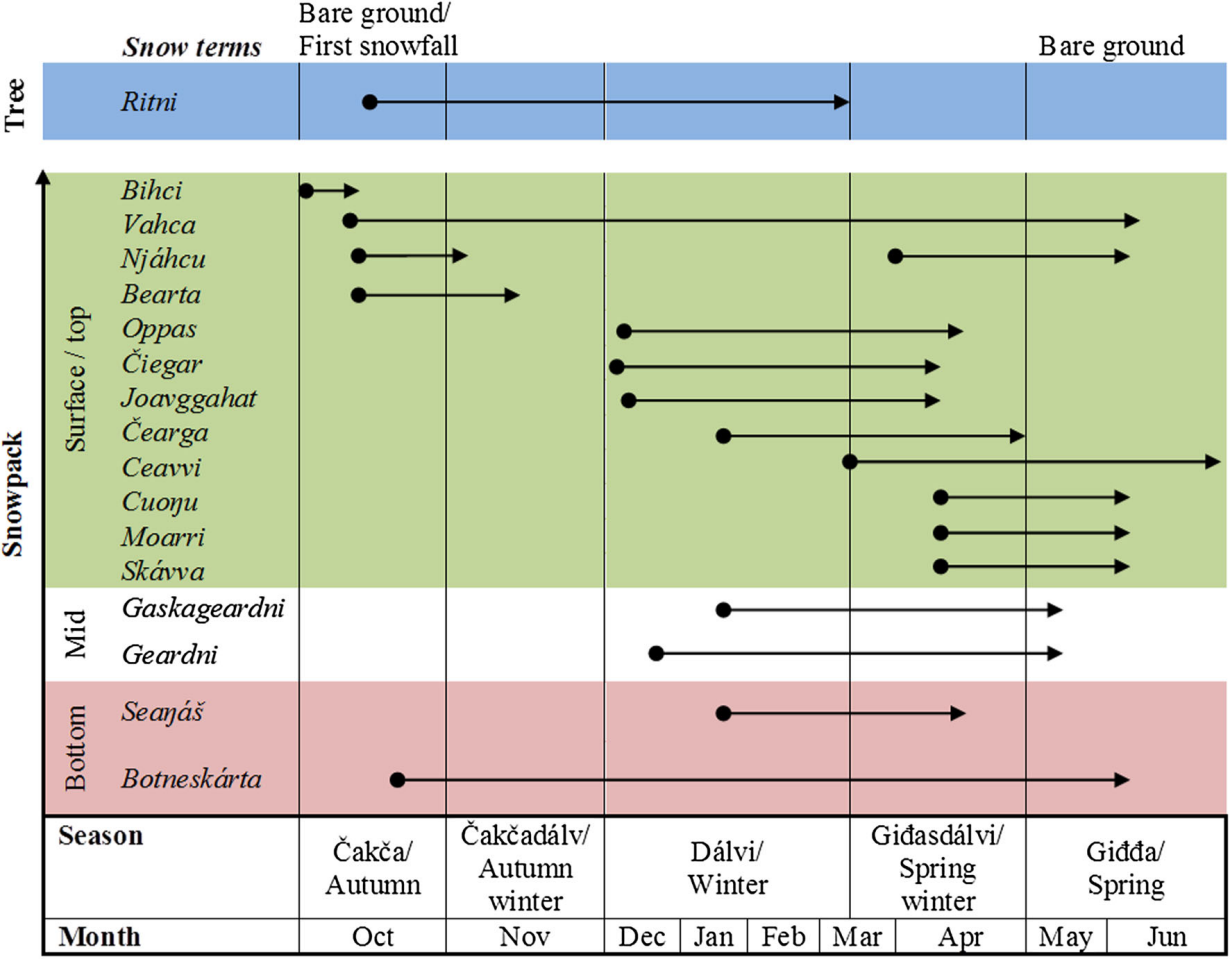
Schematic overview of Sámi snow concepts used during the cold season in reindeer herding in Guovdageaidnu, sub-Arctic Norway. The concepts are shown as they occur in and above the snowpack (*blue* frost on trees, *green* snow formation related to the surface and snowpack top layer, *white* mid snowpack layer, *pink* illustrates bottom snow layer). The *arrows* illustrate the duration of different concepts used by reindeer herders. This figure is modified from Fig. 4 by Eira et al. (2013). Further descriptions of the snow characteristics, rather than position and timing, can be found in Riseth et al. (2011)

Snow plays a central role in the cultures of indigenous Arctic people, notably for the reindeer herders of Eurasia. They have developed a holistic snow terminology integrating the effects on the ecology, grazing opportunities, and management of the herd (Fig. 4) which differs from scientific standard terms (Eira et al. 2013). However, the combination of traditional ecological knowledge (TEK) of reindeer herders with natural science measurements and snow classification may guide future strategies for a sustainable future of reindeer herding in a changing climate (Riseth et al. 2011; Eira et al. 2013). TEK in general has been formally recognized by the Arctic Council as important to understanding the Arctic (Arctic-Council 1996) and the *Ottawa traditional knowledge principles* can be found here: http://www.arcticpeoples.org/images/2015/ottradknowlprinc.pdf.

### Extreme events

Snow properties are increasingly impacted by extreme and anomalous events such as ROS (Rennert et al. 2009), icing (Bartsch et al. 2010; Hansen et al. 2013), and warming periods leading to unseasonal melt periods and isolated freeze–thaw cycles (Bokhorst et al. 2011; Semenchuk et al. 2013; Semmens et al. 2013; Wilson et al. 2013). These events are caused by different factors such as heavy rainfall (Rennert et al. 2009; Hansen et al. 2014) and movement of warm air masses through katabatic winds, e.g. Chinook (Fuller et al. 2009) and foehn winds (Pedersen et al. 2015). These extreme and anomalous events may be caused by different weather phenomena, but they all have the following in common: (1) they have an abrupt and sporadic nature, (2) they are unusual for the season in the geographical locations where they occur, (3) they cause changes in snowpack properties, and (4) they have immediate impacts on humans and ecosystems. Their temporal extent varies from a few hours to many days, and their spatial extent is controlled by the spatial scale of the driving weather phenomenon (e.g. synoptic).

The sparse distribution of meteorological stations and remoteness of areas across the Arctic region limit ground-based observation of extreme events, their effect on the snowpack, and modelling efforts (e.g. Bulygina et al. 2010; Johansson et al. 2011; Hansen et al. 2014; Pedersen et al. 2015). However, Pedersen et al. (2015) quantified the spatially distributed snow property (SWE, snow depth, snow thermal resistance, and timing of snow-free date) changes associated with episodic snowmelt events through in situ snow observations, meteorological data, and snow modelling. Extreme events are also detectable through remote sensing using differencing 3-day averages of backscatter (Bartsch et al. 2010; Semmens et al. 2013; Wilson et al. 2013). Additionally, extreme events are detectable through modelling, e.g. by Liston and Hiemstra (2011) who showed an increased trend in ROS events over maritime regions of the Arctic since 1979. Observed (Hansen et al. 2014) and predicted (Bjerke et al. 2014) abrupt changes in snow properties and snow conditions associated with extreme events add complexity to the impacts of current warming in the Arctic (Walsh 2014). Quantification and prediction of these extreme events requires increased research focus.

## Modelling changing snow conditions

### Types and applications of snow models

Terrestrial snow-cover models are used to simulate the snow temporal evolution in multiple hydrological, meteorological, climatological, glaciological, and ecological applications. Depending on the snow-model sophistication (i.e. the complexity of parameterisations used to describe snow properties and the processes taking place within the snow and at the interfaces with the atmosphere and the soil), some models can also simulate snow stratigraphy (i.e. the vertical evolution of snow properties in the various layers forming the snowpack).

Simple (empirical) snow models have been widely used in impacts studies (e.g. Van Den Broeke et al. 2010; Saloranta 2012). These models have fewer data requirements (e.g. just temperature and precipitation) than physically based models, but require calibration. For example, Kumar et al. (2013) compared the impact of using a temperature index and a physically based snow model on streamflow simulations. They found that un-calibrated temperature-index models predict streamflow poorly. Therefore, simple empirical models need to be carefully calibrated in both time and space, whereas physically based snow and hydrological models provide better accuracy. In fact, even calibrated models may be unreliable outside their regions and periods of calibration (Bougamont et al. 2007). Moreover, models based on energy balance principles are essential when snow models are required to provide boundary conditions for atmospheric models in weather and climate prediction applications and physically based snow models therefore remain essential.

Three main categories of physically based snow models exist:

- Zero-layer (combined with soil) or single-layer snow models
- Intermediate complexity snow models accounting for some physical processes within the snowpack, typically with 2–5 model layers
- Detailed snowpack models

Snow models can be driven with measured or simulated meteorological data. Usually, the higher the snow model sophistication, the simpler the framework within which they are used. There are three main configurations in which snow models are run:

- Stand-alone models
- Coupled models with atmosphere, soil, and vegetation components
- Modules within Earth System Models (ESMs)

ESMs typically use zero- and single-layer snow models because they have few parameterisations leading to fast computations, but they have limitations. Successful attempts to couple intermediate complex snow models with atmospheric and soil models have been made (e.g. within numerical weather prediction (NWP) systems and ESMs such as HTESSEL (Dutra et al. 2010), RACMO (Kuipers Munneke et al. 2011), and CLM4 (Oleson et al. 2010). Detailed snowpack models are typically used in simple stand-alone configurations. Simulation results from these models provide the temporal evolution of snow properties with depth (Vionnet et al. 2012). It is possible to drive these sophisticated models either with weather station measurements or with atmospheric reanalyses (e.g. Brun et al. 2013). A similar approach is to use coarse-grid reanalyses or climate model fields downscaled to a fine scale grid in order to account for the strong horizontal variability caused, for example, by complex orography (Fiddes and Gruber 2014). The choice of input data depends on the application, and NWP data are used for snow prediction on large scales.

Recent developments within the NWP community have resulted in increased cooperation and interests among various disciplines (e.g. hydrology and ecology). The increased spatial resolution of NWP models increases their potential utility for user groups who depend on modelling regional- and local-scale processes. This is also supported by the development of off-line land-surface models which can be run stand-alone (e.g. Crocus snow physics model).

### Progress and key achievements in Arctic snow modelling

Modelling snow cover accurately is important, particularly because of the crucial role it plays in energy transfer between the land and the atmosphere. Recent model inter-comparison projects have improved our understanding of how snow models perform and have prompted developments in individual models and parameterisations of snow processes. In this section, we highlight some achievements in snow modelling and look forward to upcoming inter-comparison experiments.

#### Snow simulation achievements and limitations

Phase 5 of the Coupled Model Inter-comparison Project (CMIP5; http://cmip-pcmdi.llnl.gov/cmip5/) provided an opportunity for assessing the simulation of snow in the current generation of climate models. Progress and limitations of CMIP5 models representing SWE, snow cover, and snowfall compared to observations and reanalyses have been identified (Brutel-Vuilmet et al. 2013; Kapnick and Delworth 2013; Terzago et al. 2014). A key result was that the decreasing trend in Northern Hemisphere spring snow-cover extent over the 1979–2005 period (Derksen et al. 2015) was underestimated by CMIP5 models (Brutel-Vuilmet et al. 2013). Snow-albedo feedbacks were modelled well but the spread in modelled snow-albedo feedback has not narrowed since CMIP3, probably due to the widely varying treatment of the masking of snow-covered surfaces by vegetation in the models (Qu and Hall 2014). Most CMIP5 models overestimate the contrast in albedo between snow-covered and snow-free land, but fewer models had large cold temperature or high snow-cover biases in CMIP5 than in CMIP3 (Fletcher et al. 2015). Because snow cover forms an interface between the atmosphere and the land surface, differences in simulations of the insulating effect of snow leads to disagreements in modelled soil temperatures (Koven et al. 2013). Representation of snow properties may also affect the accuracy of air temperature calculated by climate models. Analysis of data from 48 CMIP5 models indicates that the calculated monthly-mean surface temperature for Northern Eurasia has the largest inter-model spread during the snowmelt period indicating that accurate representation of the snowmelt is needed to improve the overall performance of models and narrow the range of associated uncertainties in climate projections.

Large sets of simulations will soon be available from climate models and ESMs in CMIP6 (http://www.wcrp-climate.org/wgcm-cmip/wgcm-cmip6) and from stand-alone land-surface models in GSWP3 (http://hydro.iis.u-tokyo.ac.jp/GSWP3/intro.html). The CliC ESM-SnowMIP project (http://www.climate-cryosphere.org/activities/targeted/esm-snowmip) has been initiated to assess the strengths and weaknesses of snow simulations in these experiments and to provide guidelines for the improvement of models.

#### Snow model forcing data

Improved simulations can result from improvements in the forcing data used to run snow models as well as from improvements in snow parameterizations. Snow-cover builds up due to solid precipitation and its properties are dramatically sensitive to liquid and mixed-phase precipitation. Though recent progress has been made (Marks et al. 2013; Mizukami et al. 2013), accurately partitioning precipitation into rain and snow remains a challenge. Multiple-year snow model forcing datasets with multiple evaluation data have recently been collated for several well-instrumented research sites in mid-latitude alpine locations (Brun et al. 2013), but there is a comparative lack of suitable data for the Arctic. For large-scale studies, global gridded forcing datasets available from reanalyses have been used successfully (e.g. Brun et al. 2013). ESM-SnowMIP includes comparisons between snow simulations at reference sites with in situ forcing data and large-scale simulations using reanalyses or coupled atmospheric models.

#### Snow parameterizations

Physical parameterizations of snow metamorphism are important because snow microstructure determines snow properties, including those controlling energy exchanges at the snow/soil and snow/air interfaces. Specific surface area (SSA) has attracted attention as a microstructural property that determines the physical, optical, and chemical properties of snow (Domine et al. 2008). It affects microwave remote sensing (e.g. Brucker et al. 2011; Roy et al. 2013; Picard et al. 2014) and it is now parameterized in some models (Carmagnola et al. 2014). SSA can now be measured in the field using observer-independent near-infrared sensors (Gallet et al. 2009; Arnaud et al. 2011; Montpetit et al. 2012). Process studies have identified weaknesses of snow models in simulating water percolation and ice-layer formation (e.g. Brucker et al. 2011; Wever et al. 2014). However, physically based snow models may help in identifying ice layers in the snow (Vikhamar-Schuler et al. 2013; Bjerke et al. 2014). Snow water mass still varies widely (50 %) among models and datasets relying solely on satellite-derived information show approximately 40 % less total snow for the peak accumulation seasons, compared with retrievals combining satellite- and ground-based data (Mudryk et al. 2015).

#### Modelling soil–snow–vegetation interactions

Forests affect snow dynamics, and models have been developed to incorporate this (Essery 2013). However, there are still issues with simulated snow-albedo feedbacks and the transition from snow-covered to snow-free canopies when temperatures rise above freezing (Thackeray et al. 2014). Shrubs trap windblown snow thereby affecting snow distribution (Myers-Smith et al. 2011) and this effect may be accentuated by the expansion of shrubs in some Arctic regions (e.g. Pearson et al. 2013; Urban et al. 2014). The impact of snow-trapping by shrubs on soil temperatures and gas fluxes have been modelled (e.g. Lawrence and Swenson 2011; Menard et al. 2014), but these processes have not yet been included in dynamic vegetation models. Progress on modelling freeze–thaw processes has been made by increasing the numbers of layers and depth of soil models, but modelling of permafrost conditions is degraded by biases in snow-depth simulations (Slater and Lawrence 2013).

#### Modelling contaminants in snow

Models now parameterize the impacts of contaminants with different spectral properties on the snow-surface albedo (Qian et al. 2015), but it remains challenging to couple these parameterisations with the atmospheric transport and deposition of contaminants such as BC. Current aerosol models can simulate mean BC concentrations in snow reasonably well, but modelled distributions are poorly correlated with measurements; models generally underestimate BC concentrations in snow in northern Russia and Norway but overestimate BC elsewhere in the Arctic (Jiao et al. 2014). Algae and bacteria living in snow and ice are also considered contaminants, and the spectral properties of snow are affected by the species composition (Lutz et al. 2014).

## Current gaps and recommendations for future research and implementation plans

Without duplicating recommendations suggested by other programmes (AMAP 2011), our intention was to review and up-date the perceived gaps in current research activities on Arctic snow changes as a contribution to the ICARP III process towards a roadmap for future research. To focus these developments, we identified key gaps, formulate recommendations, and seek commitments by stakeholders and major Arctic and Global organisations to implement these recommendations (Table 3). In addition, many detailed requirements exist which are listed in Supplementary material S1. A key limitation to progress on determining changes in Arctic snow cover and their consequences is a lack of integration among domains (land, sea, lakes, and atmosphere) and between approaches. Monitoring of snow identifies change but needs to be linked to manipulations of climate, environment, and ecosystems to understand the impacts. This understanding needs to be linked to modelling at relevant scales that project into the future (or past). With this predictive capability, knowledge-based management may be developed and implemented (Johansson et al. 2012). One possibility to improve integration of activities across domains and approaches is to develop coordinated activities, hosted by a regional or global organization.

**Table 3:** Identification of knowledge gaps related to changing Arctic snow cover and its consequences: gaps, recommendations, and implementation strategy

Gaps	Recommendations	Implementation strategy
A. Observations		
There are large *spatial scaling issues* that need to be resolved, from snow grain characteristics to the circumpolar Arctic region to the full Earth system.	(a) Increase the number of stations for manual and automatic recording (b) Develop remote sensing tools that can detect snow-depth differences across small scale landscape topography	INTERACT can provide additional measuring stations but needs information on methods and on making the data accessible GEO Cold Regions Initiative, which coordinates existing in situ and remote sensing observations of snow can facilitate, through the Global Earth System of Systems (GEOSS), data sharing and method standardization
The *temporal evolution of the Arctic snowpack* throughout an entire cold season is poorly investigated, specifically, the evolution of ice crusts and soil properties (temperature and soil frost depth)	(a) Initiate year-round ground observations are needed at intervals of hours or day (b) Improve methods to derive reliable information at a proper spatial and temporal resolution from remote sensing techniques from both optical and active (SAR) and passive (radiometer) microwave spaceborne sensors (c) Resolve technological difficulties in microwave and SAR (Synthetic Aperture Radar) remote sensing techniques	INTERACT can provide year-round measuring stations but the number and location depends on whether or not the methods are manual or remotely controlled
The Arctic is vast but is sparsely populated and *observing power is limited*	(a) Extend the number of human-based snow measurements to obtain a more detailed grid of snow parameters across the Arctic Region (b) Include citizen observations to extend the distribution of observations	
Ground-based observations of impacts of *extreme events* on the snowpack are limited	Develop detection methods (manual and remote) to quantify and record impacts on the snowpack by extreme events	
The effects of physical properties of the *snowpack on sea ice* have been measured but by out-dated methods and understanding of the snow-on-sea ice feedback is poor	(a) Improvement in the application and development of new and coordinated methodologies are required (b) Develop remote sensing techniques to quantify snowpack on sea ice	
The accuracy of *remote sensing of SWE* is limited by topography and forest cover	Develop and improve remote sensing techniques for quantification of SWE	INTERACT can provide Arctic-wide ground-validation of RS techniques over multiple topographies GEO Cold Regions Initiative can facilitate availability of remote sensing data through its Participant Organizations for inter-comparison and validation efforts
For modelling of snow precipitation, reliable measurements of *total precipitation and solid precipitation* fractions are crucial for properly driving snow models	(a) Increase the number of precipitation measuring stations to meet the needs of the modelling community (b) Equip automated weather stations with instrumentation to estimate precipitation phase—such as optical disdrometers (SPICE)	INTERACT can provide additional measuring stations but needs information on methods and on making the data accessible SPICE is evaluating current instrumentation (http://www.wmo.int/pages/prog/www/IMOP/intercomparisons/SPICE/SPICE.html)
There is great variety in methods used between different long-term measuring stations	Share and compare techniques between monitoring teams to increase the support for long-term complete validation sites with sensors probing the atmosphere, snow, and soil	INTERACT is already compiling a list of methods used at research stations and will help implement new observations and methods
B. Modelling		
The *spread of model output* needs to be reduced in relation to snow-albedo feedback, most models overestimate the contrast in albedo between snow-covered and snow-free land. Differences in simulations of the insulating effect of snow leads to disagreements in modelled soil temperatures	More accurate representation of the snowmelt is needed to improve the overall performance of the models and narrow the range of associated uncertainties in climate projections	WCRP CliC ESM-SnowMIP experiments under CMIP6 will be investigating sources of model spread in snow simulations and their influence on climate
Aerosol models can simulate mean *Black Carbon (BC)* concentrations in snow reasonably well, but modelled distributions are poorly correlated with measurements	Inclusion of particle transport from snow-free areas in GCM/regional snow models are needed and the simulation of surface albedo change due to dust deposition and microorganism growth	
Potential *feedbacks between snow and sea ice* are of critical importance, but not experimentally investigated	The snow science community urgently needs to quantify these feedbacks and include them in models if relevant	
Potential *feedbacks between snow and freshwater ice* are likely to be important because of the spatial coverage of tundra lakes and ponds. However, this has not been investigated in the field or in the laboratory while snow manipulation experiments on lake ice are absent	The snow science community needs to quantify these feedbacks and include them in models if relevant. Also, processes should be identified and quantified using experimental manipulations of snow analogues to those deployed on land	INTERACT can provide facilities around the Arctic for observations and experiments on feedbacks and for validation of models
Progress on modelling soil freeze and thaw processes has been made by increasing the numbers of layers and depth of soil models, but *modelling of permafrost conditions* is degraded by biases in snow-depth simulations	Snow-depth simulations need to be improved and coupling of snow and soil models is needed	WCRP CliC ESM-SnowMIP experiments under CMIP6 will be investigating sources of model spread in snow simulations and their influence on climate
Process studies have identified weaknesses of snow models in simulating *water percolation and ice-layer formation*	Physically based snow models may help in identifying ice layers in the snow	
*Impacts of changing snow conditions on teleconnections* within the Arctic and with other regions of Earth require more research attention	Increase the modelling effort on how changing snow conditions impact on Arctic teleconnections	
C. Impacts studies		
Effects of earlier or late snowmelt impacts on *human well-being*, such as physical injuries and degree of exposure of people to pathogens from various sources transported in snow and melt water	(a) Initiate base-line studies to assess the current threats and where in the Arctic region large changes may be expected (b) Promote research and monitoring coordination across the Arctic for inter-comparability of methodologies	INTERACT can help monitor spread of pathogens and vectors throughout the Arctic and is developing a coordinated system to do this GEO Cold Regions Initiative can provide the societal benefits assessment and awareness crossing the GEO societal benefits areas via the GEO new work programme for 2016–2025
Recent studies on *avalanche risk* assessments indicate that these may be inaccurate	Risk assessments need to be re-considered in light of changing snow conditions	
The direct impact of the temporal and spatial variability of snow on the *economic development of the Arctic*, especially expressed in monetary value, is hard to evaluate. Determining these impacts is difficult as snow conditions are changing at the same time as economic growth	Initiate an economic assessment on the cost of management and the costs associated with lack of appropriate management	
The *detailed timing of changes in snow cover during the cold season is uncertain*. These include periods of snowpack build-up, mid-winter rain events, spring snowmelt, and timing as well as increased soil moisture deficits later in the growing season	From an ecosystem perspective there is a pressing need to identify when the largest changes in snow conditions will occur, e.g., start, middle, or late winter	INTERACT can facilitate to increase the number of appropriate observations National funding agencies need to be made aware of the requirement of seasonal monitoring and experiments
*Impacts of changing snow conditions are species-specific both for plants and animals*. However, species vary in the magnitude of their contribution to key ecosystem processes	We need to identify which species are most responsive to snow changes and why, and how they will impact ecosystem processes and surface feedback to climate	INTERACT can facilitate to start appropriate observations and host relevant experiments Protocols for monitoring snow conditions and impacts in the same places and at the same scales need to be further developed in the frame of CPMP
The influences of snow and ground ice on vegetation have been investigated in some models but these processes have not yet been included in *large scale dynamic vegetation models*	Facilitate greater representation of snow-cover in all its complexity including ice layers needs to be developed in vegetation/ecosystem models	GEO Cold Regions Initiative can initiate a dedicated aim that may bridge the ecosystem mapping and snow-cover interaction
D. Linking and communicating		
*Information exchange between science and society is generally poor* with inadequate communication. Sometimes there is low relevance of the science for community needs. On the other hand, there are sometimes excessive expectations of governments on researchers and lack of understanding of science by policy makers	(a) Facilitate information exchange between society and the science community (b) Inform communities of ongoing and projected changes relevant at the local scale (c) Design observation strategies for traditional science to work together with citizens	INTERACT offers a system for communication between field researchers and local communities and has outreach activities GEO Cold regions aims to establish a proactive framework for the development of information and related services over Cold Region: the Global Cold Regions Community Portal
The Arctic science community is well integrated and coordinated by various organizations but their *agendas for research and monitoring, for example of snow cover, are often implemented independently*, even though there are numerous interactions within the Arctic and Earth systems	(a) Improve the integration between activities—monitoring, modelling, and evaluating impacts—and between Earth system domains—terrestrial, marine, atmospheric, and freshwater. (b) We need to establish archives (metadata portals) and/or a hub of in situ snow products that are relevant for the snow science disciplines and communicate awareness of the existence of these archives to other end-users (Policy makers and society)	GEO Cold Regions can help by bridging the different activities, domains, and communities (remote sensing and in situ) in the field of cold regions’ earth observations GEO Cold Regions is promoting free access to the earth observations data over the Cold Regions, including the Global Observation System of Systems (GEOSS) products and GEOSS-DataCORE

**Fig. 5:**
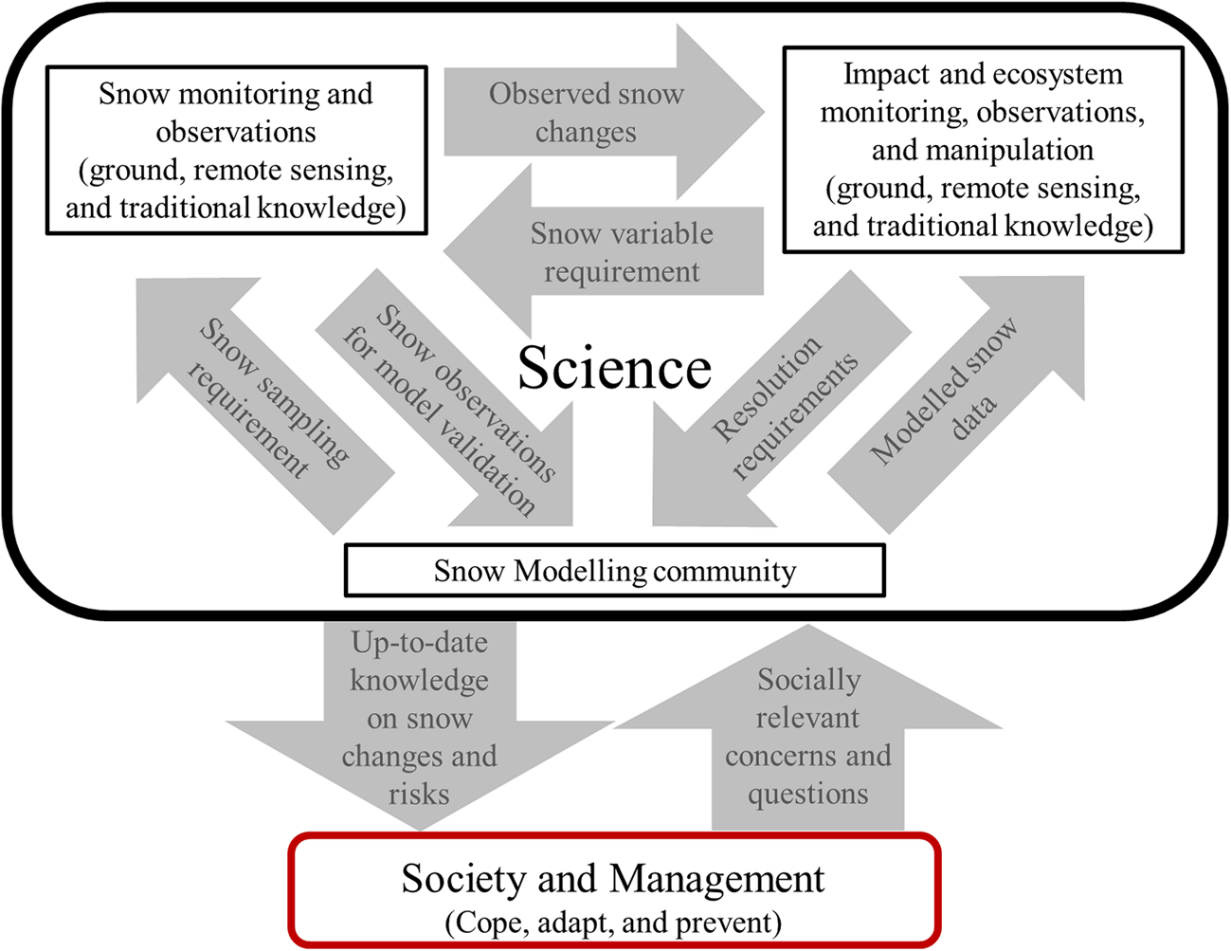
Conceptual model of required interactions between society and management and science including the snow monitoring, snow modelling, and snow-impact communities

Therefore, in order to develop ESM that can be used in the documentation and/or prediction of snow-cover changes and their impacts, there is a need for improved communication and cooperation between discipline-specific communities (ecologist/biologist, social scientists, and snow scientist) and between the approaches (monitoring/observers in the field/remote sensing and modellers) (Fig. 5). For instance, ecologists need to identify at which spatial and temporal resolutions snow-cover changes are relevant and make this known to the modelling community. This will assure that the outputs of modelled snow variables match the given resolution of ecosystem processes and dynamics. Conversely, modellers require validation data of snow variables on relevant scales (Table 1). Therefore, the timing, frequency, and spatial resolution of snow surveys and snow monitoring should match the snow-model resolution in order to generate useful snow outputs for the ecosystem scientists/snow-impact community (Fig. 5). For this interaction to be successful, detailed cross-disciplinary coordination of field campaigns, monitoring, research projects, and model development is required.

Since society and its infrastructure have to cope with the challenges of changing snow conditions (Fig. 1), it requires easy access to snow predictions. Therefore, an open dialogue needs to be established or expanded to facilitate information exchange between society and the science community. Implementation of these recommendations should ideally be considered by organizations, such as the Arctic Council, that span science and human dimensions. Integration between the different snow disciplines and communication to end-users could be achieved through the ICARP process and associated organizations IASC, INTERACT, CliC, GEO (GEOSS), and WMO (GCW). With this paper, we have attempted to provide a basis, and stimulus, for the implementation of key priorities (Table 3) to address the limitations in our understanding of Arctic snow conditions and how they may change in the near future.

## Electronic supplementary material

Below is the link to the electronic supplementary material.

Supplementary material 1 (PDF 44 kb)
