# Supplementary material for: Changing Arctic snow cover: A review of recent developments and assessment of future needs for observations, modelling, and impacts
Source: Ambio. 2016 Mar 17;45(5):516–37. doi: 10.1007/s13280-016-0770-0 (PMC4980315; doi:10.1007/s13280-016-0770-0)
Supplement: Supplementary file 1 — Supplementary material 1 (PDF 44 kb) [file 13280_2016_770_MOESM1_ESM.pdf]

Ambio

Electronic Supplementary Material

This supplementary material has not been peer reviewed.

Title: Changing Arctic snow cover: a review of recent developments and assessment of future needs for observations, modelling and impacts

Authors: Stef Bokhorst, Stine Højlund Pedersen, Ludovic Brucker, Richard Essery, Oleg Anisimov, Jarle Werner Bjerke, Ross Brown, Dorothee Ehrich, Achim Heilig, Niila Inga Leavas, Susanne Ingvander, Cecilia Johansson, Margareta Johansson, Ingibjörg Svala Jónsdóttir, Giovanni Macelloni, Heather Mariash, Donald Mclennan, Ninis Rosqvist, Atsushi Sato, Hannele Savela, Martin Schneebeli, Aleksandr Sokolov, Sergey A Sokratov, Silvia Terzago, Dagrun Vikhamar-Schuler, Scott N. Williamson, Yubao Qiu, and Terry V. Callaghan

SI: Detailed recommendations for future focus on snow science

1. Issues specific to the snow observation community

Ground based observations

1) Standardize the snow observations by using current knowledge/guidelines/ongoing standardizing efforts. (through: GCW CryoNet, WMO SPICE)
2) Improve methods to quantify the interplay between the impacts of changing temperatures and snow.
3) Integrate methods operating at different scales by upscaling and downscaling observations. (through GCW CryoNet)
4) Identify causes of differences between remote sensing products. (first initiation by SnowPEX)
5) Develop non-subjective instrumentation (at a reasonable price) to fill gap in current observational efforts, e.g. SWE, density, specific surface area, and liquid water content. Utilize existing cross-comparison and testing facilities for validation.
6) Increased research focus related to snow on ice (sea, lakes, and rivers).
7) Improve observations of snowfall, precipitation phase and methods for correcting observed snowfall during windy conditions. (WMO SPICE)
8) Adapt monitoring methods to identify and monitor events making use of citizen science.

Remote sensing

9) Identify methods to reduce cloud-cover contamination at small spatial scales relevant to ecosystem processes and infrastructures.
10) Integrate scales of RS coverage by upscaling and downscaling, for example by improving snow-related satellite data (snow products) at the watershed level.
11) Improve methods to quantify snow mass on land and ice.

12) Convert backscatter observations into more accurate SWE retrievals and explore application of remote sensing SWE retrievals in the microwave domain beyond flat areas to mountains.
13) Improve methods to measure snowpack stratigraphy (ice layer, rain-on-snow, metamorphism).
14) Adapt monitoring methods to identify and quantify extreme-unusual events during the cold season (without interfering with long-term monitoring records).
15) Standardise the RS snow observations by using current knowledge/guidelines/ongoing standardizing efforts.
16) Increase data gathering on SWE, especially for areas like Central Asia, Tibetan Plateau, and Mongolia.

2. Issues specific to the modelling community

Overall, identify key snow processes that need to be implemented in ESM because all the parameterisations in state-of-the-art snow models cannot be considered (too time consuming, very expensive, and predictive power is reduced).

Recommendations in relation to scaling

1) Snow processes in global climate models are only coarsely represented but evaluating the complex interactions between snow and climate requires finer spatial resolutions, especially in complex topography. It is necessary to bridge the scale gap between the global and local scales by pursuing developments of downscaling methods, and afterwards to evaluate how the uncertainties in simulated variables propagate from large to small scales.
2) Determine the role of snow as the driver of changes on other Earth's system components.
3) Assess the role of snow in regional responses to climate forcing (e.g. Arctic amplification), focusing on feedbacks in historical and future scenario runs.
4) Refine the parameterisations of processes in the snow-covered soil (e.g. changes in depth of active layer). Snow cover spatial heterogeneity matters significantly for the control of soil properties, which motivates the need for developing further the probabilistic models.
5) Improve SWE simulations for river runoff.
6) Improve the accuracy on drifting snow parameterisation.

Recommendations on specific modelling issues

7) Evaluate the representation of extreme-unusual events in models.
8) Apply data assimilation techniques of passive microwave observation in sophisticated multi-layer snow evolution models in operational practice (at the large scale).
9) Refine snow simulation in forested areas, because of the importance of the snow-albedo feedback.
10) Couple aerosol transport and deposition models with snow models to evaluate the importance on snow-covered surfaces (e.g. forest fire impacts on the Greenland Ice Sheet).
11) Include the snow chemistry parameterisations in the ESM.
12) Include findings of the evolution of the snowpack, i.e. changing grain forms, in the parameterisation of snow models.
13) Assess the contribution of snow cover anomalies to current and future predictability of regional temperature and precipitation patterns.

14) Attribute differences in snow model performance to differences in snow model physics, calibration, and numerics using stand-alone simulations (gridded data and well-instrumented reference sites).
15) Identify differences in simulations due to differences in representations of interactions between snow, soil, and vegetation.
16) Model products could be another way for data expansion in spatial-temporal coverage.
17) Develop accurate simulations of temporal and spatial variability in snow depth and SWE to drive hydrological models, improve simulations of river runoff, and thus improve water resources management. Tools with a fine spatial resolution will have to be provided to investigate how increasing temperatures will affect future solid precipitation, snowpack, river runoff, and water availability.

Recommendations concerning factors interacting with snow properties

18) Introduce the **black carbon** (BC) contribution in parameterisations of snow albedo to allow a better representation of snow-climate interactions.
19) Develop accurate parameterisations of **sublimation** from drifting snow and generation of subgrid snow-depth variations.
20) Improve the representation of snow-vegetation interactions through mechanical impacts (load/relief) and difference in energy conductance in snow models.

Recommendations for the coupling of sophisticated snow models with other systems

21) Develop multi-layered snow evolution models simulating the snow stratigraphy (in contrast to one- or two-layer snow models) because ESM users and other user groups (e.g. studying hydrology, ecology, vegetation) recognize the benefits of this approach.
22) Evaluate CMIP5 results in the specific context of their skills in the Arctic and "end model democracy".
23) Implement an intercomparison of snow simulations within the CMIP6-Endorsed Land Surface, Snow and Soil Moisture MIP (LS3MIP).
24) Identify differences in simulations due to differences in representations of interactions between snow, soil and vegetation.
25) Improve parameterisations of processes in snow-covered soil (e.g. changes in depth of active layer), and move from deterministic to probabilistic models. Spatial heterogeneity in snow cover matters significantly for the control of soil properties, which motivates the need for further developing probabilistic models.
26) Identify key snow processes that need to be implemented in ESM; not all the parameterisations in state-of-the-art snow models can be considered (too time consuming, very expensive, and reduction in predictive power).
27) Couple aerosol transport and deposition models with snow models to evaluate the effect of absorbed aerosols on snow albedo (e.g. forest fire impacts on the Greenland ice sheet).
28) Include snow chemistry parameterisations in ESMs.
29) Improve understanding of the role of snow in climate feedbacks.
30) Improve understanding of the role of snow as a driver of changes in other Earth system components and as a driver of environmental changes in the Arctic.
31) Improve understanding of transitions between snow and rain to support the study of the question of whether more of the precipitation will fall as rain or as snow in future climates.

32) Assimilate snow depth and extent observations used operationally in NWP models with simple snow schemes (one-layer models), and develop data assimilation techniques for the more sophisticated snow models and passive microwave observations (see for instance: SnoDAS, and CalDAS)
33) Further develop and test multi-layer snow models to study extreme events and their environmental and socioeconomic impacts.
34) Assess the contribution of snow-cover anomalies to current and future predictability of regional temperature and precipitation patterns.
35) Improve quantification of socioeconomic impacts by determining the interplays between the magnitude and rate of snow changes, between changes in snow and changes in temperature and between the projected increase in precipitation and the shorter snow period.

3. Issues specific to understanding the impacts of changing snow conditions on ecosystems and societies

1) Identify environmental human health and socio-economic consequences of extreme snow events and conditions, increased precipitation and the magnitude and rate of snow changes.
2) Identify potential links between marine, terrestrial, and freshwater ecosystems through the cold season snow.
3) Implement monitoring of episodic climate events in the existing Arctic monitoring stations, and develop citizen science to register relevant observations.
4) Develop a more detailed understanding of how topographical features, vegetation structure, and weather spatio-temporal variability in snowpack properties at scales relevant to subnivean organisms.
5) Quantify the role of (changing) snow conditions on the distribution of pathogens and harmful chemicals.
6) Determine how snow conditions affect agricultural practices and availability of drinking water (snowmelt is a major water resource for many communities in summer).
7) Determine how the carbon flux from terrestrial ecosystems is affected by the duration, thickness, and properties of the snow cover.
8) When are snow conditions changing during the cold period? When can we expect major shifts in snow conditions (such as spatial and temporal spread in snow deposition and melting rates)?
9) Include cold season weather processes in modelling of Arctic biomass trends ("the greening of the Arctic")
10) Evaluate the effects of cold season climate change on Arctic biodiversity, with particular focus on keystone species and the extinction risk of threatened species.
11) Assess the role of snow (e.g., winter snow cover, snowmelt flooding effects) on the distribution, composition and productivity of Arctic terrestrial communities, and the subsequent effects of snow change on factors such as their value as habitat, their C flux dynamics, and their feedbacks to earth climate systems.
